# Evidences of inner Se ordering in topological insulator PbBi_2_Te_4_-PbBi_2_Se_4_-PbSb_2_Se_4_ solid solutions

**DOI:** 10.1038/s41598-020-64742-6

**Published:** 2020-05-14

**Authors:** Yuya Hattori, Yuki Tokumoto, Koji Kimoto, Keiichi Edagawa

**Affiliations:** 1 Institute of Industrial Science, The University of Tokyo, Komaba, Meguro-ku, Tokyo, 153-8505 Japan; 2 National Institute for Materials Science (NIMS), Tsukuba, Ibaraki, 305-0044 Japan

**Keywords:** Materials science, Physics

## Abstract

In topological insulators (TIs), carriers originating from non-stoichiometric defects hamper bulk insulation. In (Bi,Sb)_2_(Te,Se)_3_ TIs (BSTS TIs), however, Se atoms strongly prefer specific atomic sites in the crystal structure (Se ordering), and this ordering structure suppresses the formation of point defects and contributes to bulk insulation. It has accelerated the understanding of TIs’ surface electron properties and device application. In this study, we select Pb(Bi,Sb)_2_(Te,Se)_4_ (Pb-BSTS) TIs, which are reported to have larger bandgap compared to counterpart compound BSTS TIs. The Se ordering geometry was investigated by combining state-of-the-art scanning transmission electron microscopy and powder X-ray diffractometry. We demonstrated the existence of inner Se ordering in PbBi_2_(Te,Se)_4_ and also in Pb-BSTS TIs. Quantitative analysis of Se ordering and a qualitative view of atomic non-stoichiometry such as point defects are also presented. Pb-BSTS TIs’ Se ordering structure and their large gap nature has the great potential to achieve more bulk insulation than conventional BSTS TIs.

## Introduction

In 1980, the Quantum Hall Effect (QHE) was first discovered by Klitzing *et al*.^1^. In the QHE system, the peculiar topology of band structure yields boundary states called skipping edge states in which backscattering is completely prohibited^2^. Since then, the importance of the band structure topology has come to be recognized^3,4^. The drawback of the QHE system is that strong magnetic field is required to generate such special states, which hampers its device application. However in 2005, the existence of such boundary states, even in zero magnetic field, was theoretically predicted^5^. This was confirmed experimentally by transport analyses and angle-resolved photoemission spectroscopy (ARPES) studies soon after its prediction^6–8^. This first developed material group possessing such boundary states is called topological insulators (TIs)^9,10^. The TIs’ surface states are an extension of 1D edge skipping electron in QHE^2^, and the surface states have exotic properties such as suppression of backscattering^11,12^ and spin polarized nature^13^. By using the spin polarized current^14^, spintronics devices with considerably lower power consumption can be obtained^15^. Intensive efforts for application have been made both from theoretically^16^ and experimentally^15^. In particular, Mellnik *et al*.^15^ succeeded in exerting a strong spin-transfer torque from the TI layer to an adjacent ferromagnetic layer, and changing the direction of magnetization in the ferromagnetic layer. This mechanism can be utilized in ultralow power logic technologies^17^ in next generation.

In the TIs field, tetradymite-type Bi_2_(Te,Se)_3_ (BTS TIs) and (Bi,Sb)_2_(Te,Se)_3_ TI (BSTS TIs) have often been used for detecting and utilizing specific surface states^18–21^. In these materials, Se atoms predominantly occupy the center site of a quintuple(5)-layer structure (Se ordering)^22^, and are believed to suppress the formation of antisite defects^21^. The mechanism is as follows^21^: (i) the concentration of Se vacancy is reduced because the Se trapped between two Bi atoms is less exposed to evaporation due to stronger chemical bonding with Bi; (ii) antisite defects between Te and Bi is expected to be suppressed because of preferable bonding between Se-Bi compared to Se-Te bonding; (iii)ordered nature minimizes the additional disorder that could be caused by Se/Te randomness. In addition to Se ordering, chemical potential tuning by changing Bi/Sb ratio makes it possible to enhance the BSTSs’ bulk insulation to more than 1000 times higher than the non-doped sample^20,21^. These efforts allow one to verify the 2D nature of surface states, and to detect its π Berry phase^23^ and other important properties such as mobility by transport measurements^18,20,21,24^. However, even in such ideal samples, the utilization and detection of surface states remain a bit difficult for now. Therefore, many research groups search for new materials that have ideal properties, and as such Pb-based TIs (Pb-TIs) were rediscovered in the context of solid-state physics.

Pb(Bi,Sb)_2_Te_4_ is one of the Pb-TIs. It was theoretically predicted as TI in 2011^25,26^, and experimentally verified in March^27^ and May^28^ of 2012 by two different groups, which demonstrates the intense competition in this field. Kuroda *et al*.^28^ showed that this material has more than twice larger density of surface electron than BSTS TIs when compared at the same Fermi energy from the Dirac point. This property makes Pb-TIs suitable candidates for spintronics research and application. Also, it is reported that this material belongs to a peculiar topological class (*v*_0_; *v*_1_*v*_2_*v*_3_) = (1; 111)^25,28^, while most of the other TIs like BSTS have (1;000)^29^. These nonzero indices originate from band inversion at the Z point; TIs with such indices are predicted to possibly have gapless dislocation states^30,31^. In the study by Souma *et al*.^27^, it was shown that the chemical potential of Pb(Bi,Sb)_2_Te_4_ can be tuned between the bulk band gap, and that it is always a strong TI. In response to these studies, the authors synthesized various Sb-concentration samples with fine Sb tuning, and succeeded in enhancing the bulk insulation approximately 400 times higher than non-doped one^32,33^. Its remaining bulk conduction was considered to come from impurity bands, and the magnetoresistance was quite well consistent with 3D-weak antilocalization scheme. Therefore, there should be random potential possibly coming from point defects and also local band-bending should occur. In such circumstances, an effective band gap *E*_*eff*_, which is the activation energy in transport measurements, will become smaller^34,35^. The theoretical study has revealed *E*_*eff*_ will become *E*_*eff*_ = 0.15Δ*E*_*g*_ in ordinal case^34^, which is consistent with activated energy in transport measurements^20,21,32^. To suppress the formation of such point defects and local band-bending, we began to focus on the Se ordering structure.

**Figure 1:**
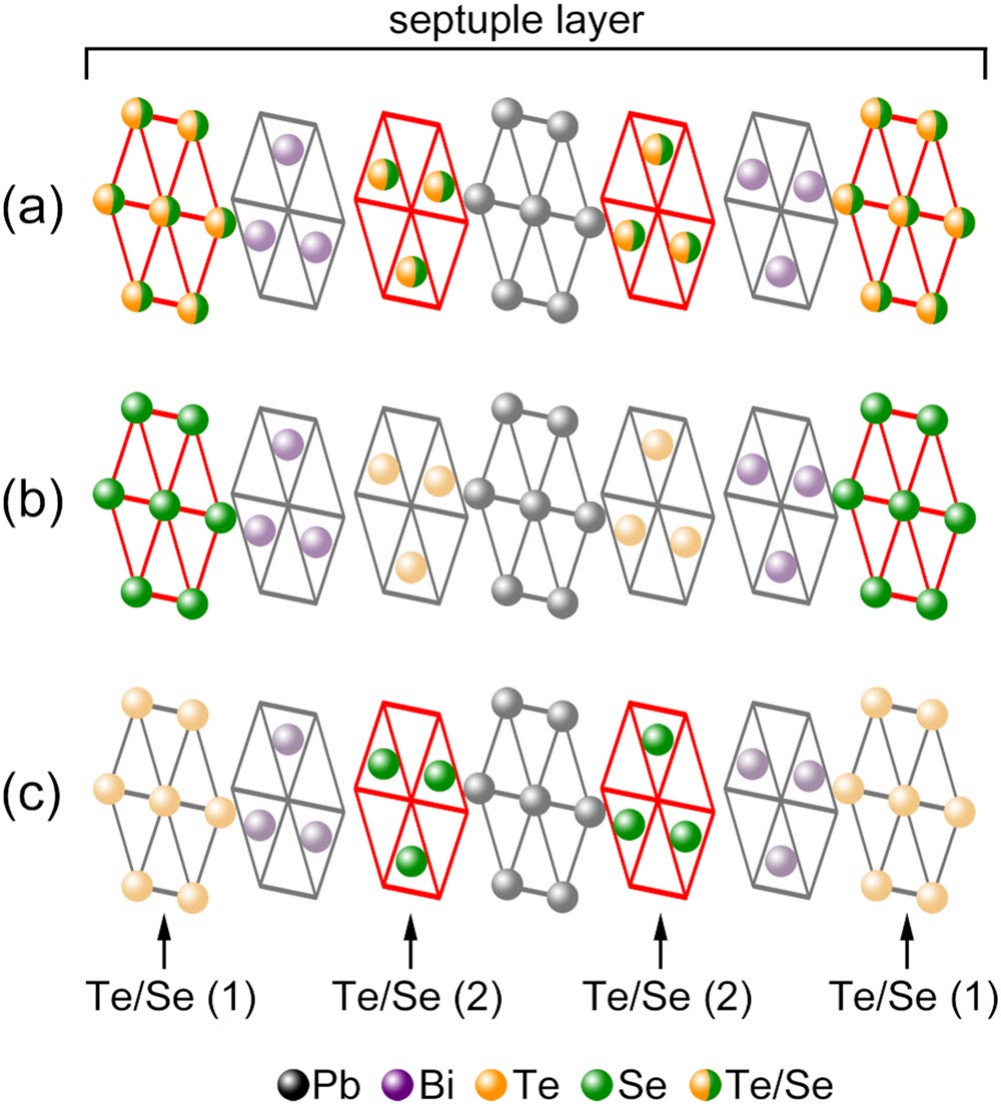
Possible Se geometry in PbBi_2_(Te,Se)_4_: (**a**) disordered Se occupancy; (**b**) outer Se ordering; and (**c**) inner Se ordering. Se atoms are emphasized as a green sphere. Se atoms occupy the outer Te/Se(1) sites or inner Te/Se(2) ones.

The substitution effect of Te by Se atoms in PbBi_2_Te_4_ was studied by some groups^36–38^. This material was first investigated in the 1970s as a natural mineral “poubaite”^36^, and it was found that its crystal structure is the same as that of PbBi_2_Te_4_^36^. However, detailed crystal structure, such as site occupancy and the Se ordering structure, was not determined due to tiny crystal size (approximately 30 *μ*m). After theoretical prediction as TI, the nanosheet of PbBi_2_Se_4_ was synthesized by Chatterjee *et al*.^37^ although the bulk sample of PbBi_2_Se_4_ with the crystal structure of $R\mathop{3}\limits^{-}$ is unstable^39^. The observed band gap is 600 meV, which is considerably higher than that of counterpart compound Bi_2_Se_3_ (300 meV). As for theoretical research, the effect of the Se distribution geometry in PbBi_2_(Te,Se)_4_ was studied in 2017^38^. It was reported that PbBi_2_(Te,Se)_4_ (Pb-BTS) can take 3 different Se geometry structures (Fig. 1a–c): (a) disordered; (b) outer Se ordering; and (c) inner Se ordering. They showed that the electronic structure strongly depends on Se atom geometry, and that the maximum band gap can be obtained in geometry (c). Considering the large bandgap^37,38^ and the defect suppressing nature of Se ordering^21^, it is expected that significantly more bulk insulation samples can be obtained if the inner Se ordering geometry really occurs in Pb-TIs. The bulk insulation strategy is the same as in BSTS TIs; however, larger bandgap has more potential to achieve better bulk insulation than BSTS TIs.

In this study, by combining state-of-the-art scanning transmission electron microscopy (STEM) and powder X-ray diffractometry (pXRD), we studied the Se distribution geometry in PbBi_2_(Te,Se)_4_. In the STEM study, we directly observed the inner Se ordering in PbBi_2_(Te,Se)_4_ (Pb-BTS) TI. The pXRD study also revealed that the strong tendency of Se atoms to occupy the inner site, and 90–100% of Se atoms are considered to occupy inner Te/Se(2) site. Also, we discovered that this ordering also occurs in Pb(Bi,Sb)_2_(Te,Se)_4_ (Pb-BSTS), which enables the bulk insulation by Fermi level tuning. A qualitative view of atomic non-stoichiometry such as point defects are also presented. These findings pave the pathway to develop a new TI material group possessing more bulk insulation than that of the conventional BSTS TIs.

## Results and Discussions

### Phase constitution of PbBi_2_(Te_1-y_Se_y_)_4_ and Pb(Bi_1-*x*_Sb_*x*_)_2_(Te_1-*y*_Se_*y*_)_4_

**Figure 2:**
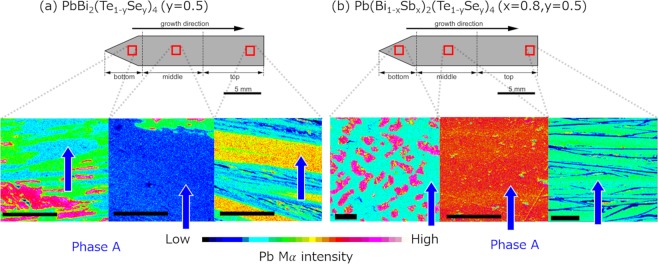
EPMA Pb mapping of (**a**) PbBi_2_(Te_1-y_Se_y_)_4_ alloy system (y = 0.5); and (**b**) Pb(Bi_1-x_Sb_x_)_2_(Te_1-y_Se_y_)_4_ (x = 0.8, y = 0.5) system. The colormap is normalized for each picture. The length of black bar in all pictures is 200 μm. The region of the target compound Pb(Bi,Sb)_2_(Te,Se)_4_ is indicated by blue arrows.

The phase constitution and composition of the grown crystals were investigated by an electron probe microanalyzer (EPMA). In Fig. 2a, EPMA mapping of Pb-BTS (*y* = 0.5) is presented. Detailed information of characteristic X-ray is presented in the Supplementary Information. Phase PbBi_2_(Te_1-y_Se_y_)_4_ (phase A) is indicated by blue arrows, and a detailed composition is listed in the Supplementary Information (S1). Phase A is always with other impurity phases Pb_5_Bi_6_(Te,Se)_14_ and PbTe. That is possibly due to complex phase diagram in PbSe-Bi_2_Se_3_ series compound, which includes several peritectic reactions^39^. In our synthesized rod, Pb_5_Bi_6_(Te,Se)_14_ seems to be originating from a peritectic reaction of PbTe + melt → Pb_5_Bi_6_(Te,Se)_14_. Contrastingly, Fig. 2b shows the EPMA-Pb mapping of Pb(Bi_1-x_Sb_x_)_2_(Te_1-y_Se_y_)_4_ sample (*x* = 0.8, *y* = 0.5). The phase distribution completely changes compared to Pb-BTS, and its microstructure is similar to that observed in Pb(Bi_1-*x*_Sb_*x*_)_2_Te_4_ (*x* = 0.6–0.9) Bridgman rod^32^. Single phase of Pb(Bi_1-*x*_Sb_*x*_)_2_(Te_1-*y*_Se_*y*_)_4_ (phase A’) can be easily picked out, which is good condition for the transport study both for bulk samples and nano exfoliated samples.

### STEM observations

**Figure 3:**
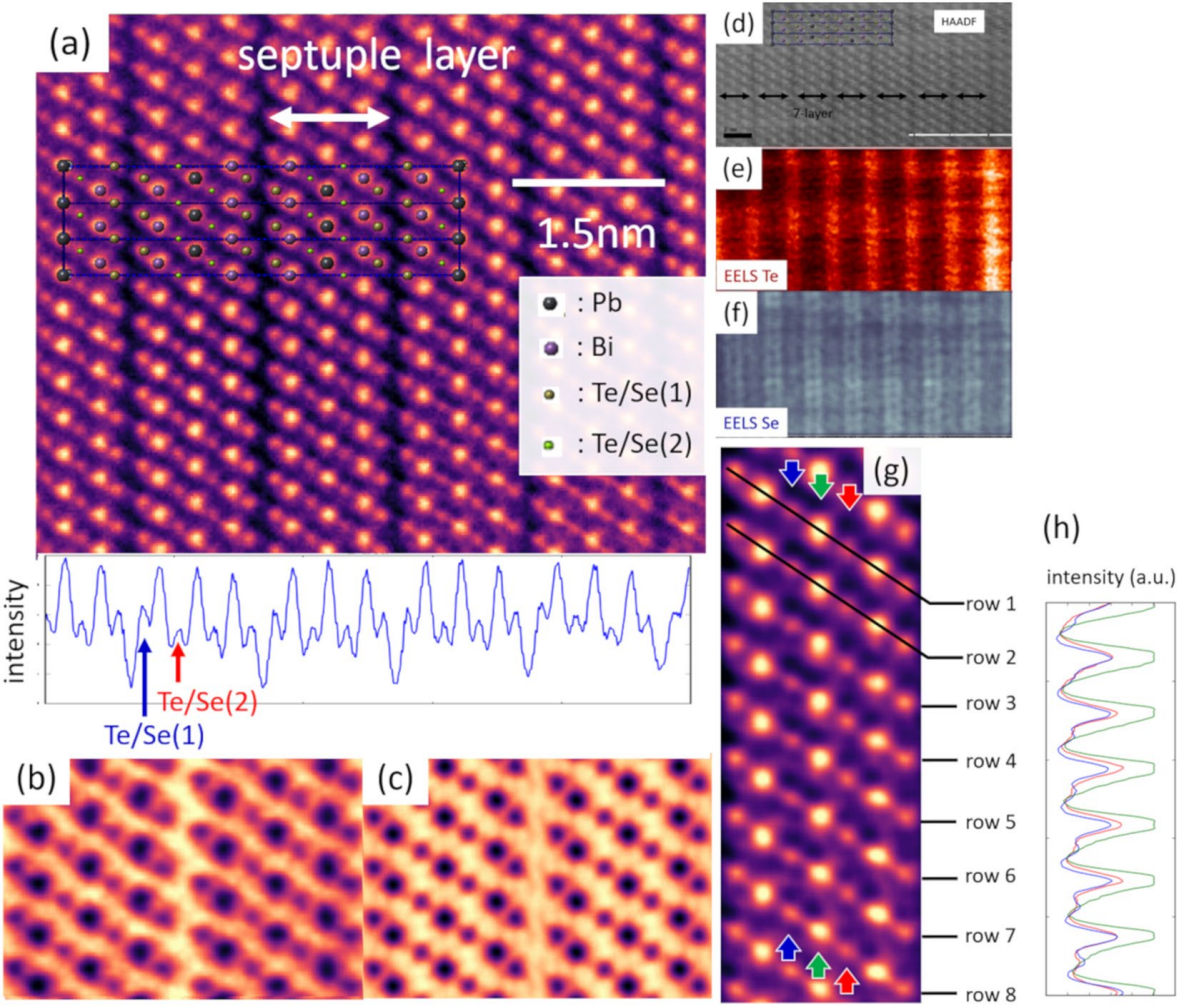
(**a**) HAADF raw image with intensity plot of the sample of PbBi_2_(Te_1-y_Se_y_)_4._ (y = 0.5) Structure model is also presented; (**b**) experimental ABF-image (RL deconvoluted); and (**c**) simulated image of 100% inner ordered PbBi_2_(Te_1-y_Se_y_)_4_ (y = 0.5); (**d**) HAADF-STEM; (**e**) EELS-Te mapping; and (**f**) EELS-Se mapping in the same area; (**g**) magnified HAADF image with intensity profile; (**h**) intensity is plotted along the blue, green, and red arrows.

A cross-sectional high-angle annular dark field (HAADF) - STEM image of PbBi_2_(Te_1-y_Se_y_)_4_ (*y* = 0.5) along [100] direction together with intensity profile is presented in Fig. 3a. This image is a drift corrected image using in-house software^40^. The unique septuple (7) layer structures are clearly observed and the thickness of one unit of a septuple layer is estimated to be 1.38 nm, which is in quite good agreement with the previous XRD crystal structure analyses results^38^. The intensity of the HAADF image is assumed to be proportional to *Z*^2^ in an ordinal condition^41,42^. From the intensity profile below, we can clearly see the inner Te/Se sites (Te/Se(2)) have weaker intensity than the outer Te/Se(1) sites. As the atomic number of Se (*Z* = 34) is less than Te (*Z* = 52), it is safe to assume that Se atoms mainly occupy the inner Te/Se(2) sites.

In Fig. 3b, the annular bright-field (ABF)-STEM image of the same sample is presented. This image is processed by Richardson-Lucy software^43^ for resolution enhancement and noise reduction. The validity of denoising process is examined in the Supplementary Information, in which we compare deconvoluted image with raw image (S2). Briefly, ABF and HAADF-STEM images are produced by the convolution of scattering function of atomic column and the probe intensity profile of STEM system under the incoherent imaging approximation^41^. Therefore, to enhance the resolution of STEM image, the algorithm using deconvolution process, such as Richardson-Lucy (RL) deconvolution, is a powerful tool. In fact, Ishizuka *et al*.^44^ utilized these algorithms to enhance the spatial resolution in HAADF and energy resolution in EELS. We also tried the ABF-STEM image simulation (Fig. 3c) with a somewhat extreme condition: Te/Se(1): 100%Te, Te/Se(2): 100% Se. The simulated image is quite similar to the experimental one; thus the occupancy of the inner Te/Se(2) sites might be near 100% Se. We also admit that it is somewhat risky to determine site occupancy only from this image because of the background noise coming from the dechanneling effect; therefore, quantitative analysis on site occupancy was conducted by pXRD analyses and is discussed later.

Other than HAADF and ABF-STEM, recent breakthrough of STEM-EELS has led to finding ideal experimental conditions to suppress delocalization of probe electrons^45,46^, and atomic-resolution chemical mapping and analyses of atoms’ electron state have become possible^45^. Figure 3d–f were taken in the same area simultaneously, and they are HAADF-image (Fig. 3d), EELS chemical mapping of Te M_4,5_ edge (572 eV, Fig. 3e), and Se L_2_, L_3_ edges (1436 eV, Fig. 3f). The black arrows in Fig. 3d are single septuple layer. The Te intensity between septuple layer, that is Te/Se(1) sites, is high (Fig. 3e). Correspondingly, the Se intensity in the septuple layer (Te/Se(2)) is high (Fig. 3f). These facts also strongly suggest the inner Se ordering. Therefore, from all of the experimental results of HAADF, ABF-STEM, and STEM-EELS, we can conclude that the inner Se ordering actually occurs among three possible Se geometries^38^.

In addition to Se ordering, some information about local non-stoichiometry can be deduced from the STEM observation. In the previous paragraph, we showed that inner Se ordering occurs and Se occupancy in the Se layer might be nearly 100%, but this is an average structure. When we pay attention to the local structure, this outlook should be slightly altered. Figure 3g is taken in the same TEM sample of 3a. This image is also processed by RL software (for the validity, see the Supplementary Information S2). Although the intensity of Te, Bi, and Pb sites hardly changes in the image (see the equivalent peak intensity on the green line of Fig. 3h), there is an intensity fluctuation in the Se(2) layer (Fig. 3h). In some points, high intensity compatible to the outer Te layer is observed (row 4, 5 red). Also, they appear to form pairs with low intensity columns (row 4,5 blue) across the middle Pb sites (green arrow). Such intensity fluctuation might come from atomic level local non-stoichiometry. Such local non-stoichiometry cannot be measured by conventional XRD analyses, which yield the average structure. STM measurements also give information only about outermost layer of exfoliation layer. However, recent STEM technique allows one to obtain the information of atomic level local non-stoichiometry deep under topmost cleavage plane. For example, in the compound BAs, STEM study revealed that As_B_ and B_As_ form an antisite pair locally^47^ and proposed as the reason of low thermal Conductivity of this material. As for the local non-stoichiometry in Fig. 3g, the Te_Se_ & Se_Te_ antisite pair^48^ and the V_Se_ & Bi_Se_ antisite^49^ pair might be the main candidates. In BSTS TIs, the Te_Se_ and Se_Te_ antisites defects are shown to be quite stable under almost all growth conditions^48^. The V_Se_ & Bi_Se_ antisite pair is actually observed in Bi_2_Se_3_ by STEM observation^49^. In any case, such local non-stoichiometry is considered to cause local band-bending^34,35^ and charge puddles are generated^35,50^. More study is needed to elucidate such point defects nature, which is the key factor to enhance bulk insulation in TIs and understand its nature.

### Quantitative analyses of Se occupancy by pXRD

**Figure 4:**
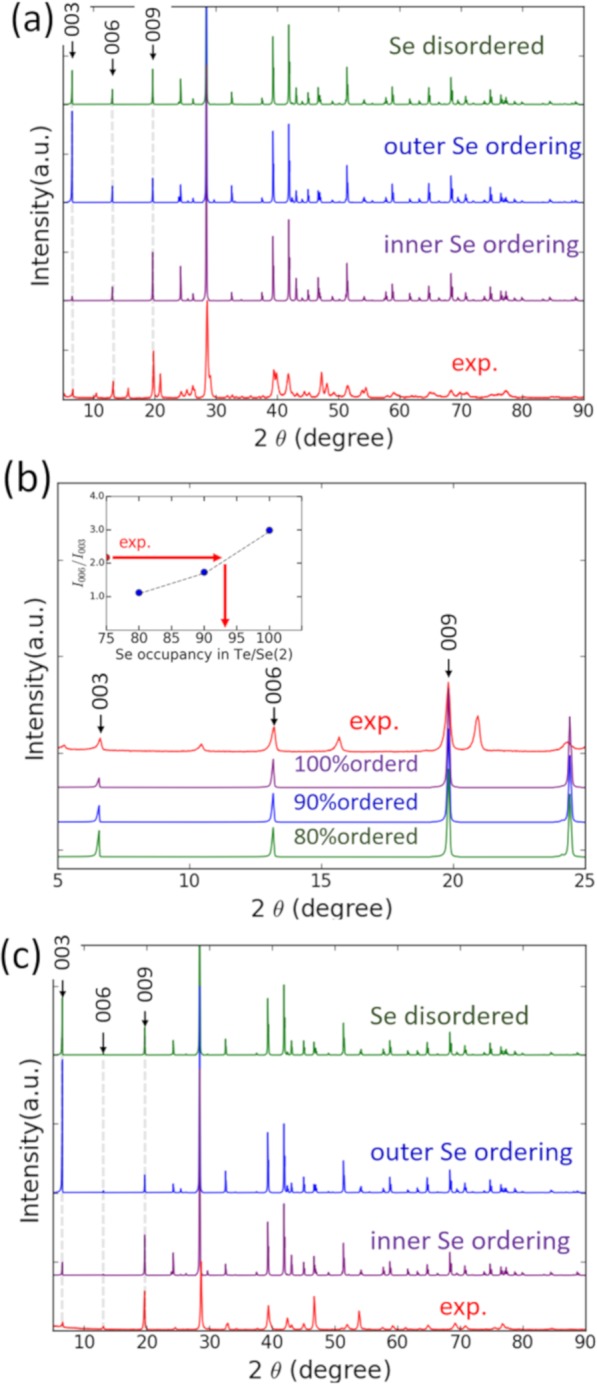
(**a**) The simulated pXRD patterns of 3 different Se geometries and the experimental pXRD data of PbBi_2_(Te_1-y_Se_y_)_4_ (y = 0.5); (**b**) pXRD simulation patterns with different Se ordering degree are presented with experimental data of PbBi_2_(Te_1-y_Se_y_)_4_ (y = 0.5); (**c**) pXRD simulation patterns of different Se ordering in Pb(Bi_1-x_Sb_x_)_2_(Te_1-y_Se_y_)_4_ (x = 0.8, y = 0.5) are presented with experimental data.

The quantitative study of Se occupancy of Te/Se(1) and Te/Se(2) sites by pXRD is presented in this section. In pXRD measurements, a small DS slit angle was used to accurately evaluate the pXRD intensity in the lower 2 θ angle. The pXRD simulations were conducted using RIETAN-FP^51^. In Fig. 4a, the simulated pXRD data of three possible Se geometry: (1) disordered Se; (2) outer Se ordering; and (3) inner Se ordering are presented with experimental data. It should be noted here that we have a strong preferred orientation (PO) in our powder sample due to van der Waals cleavage nature of PbBi_2_(Te,Se)_4_ in parallel with (00h) plane. This inhibits a simple comparison of the experimental data with the simulated ones. Nevertheless, we should be allowed to make such comparison if we focus on a series of 00h peaks, because they should hold correct intensity ratios even if the PO effect is strong. In the simulated data, we can see that the intensity ratio of 003 and 006 strongly depends on the Se geometries. This originates from layer stacking order in the [001] direction and can be used for the geometry determination. Other factors that can affect the intensity ratio of 00h reflections are investigated in the Supplementary Information S3, and they are shown to give much less contribution than Se geometry. In the experimental data, we notice the trace of an impurity phase, which is identified as phase Pb_5_Bi_6_(Te,Se)_14_. However, if we compare 00h reflections of PbBi_2_(Te,Se)_4_, then the experimental data most fit the inner Se ordering simulation, which is consistent with earlier STEM results. In order to investigate Se occupancy quantitatively, we then set the occupancy of the Te/Se(2) sites as (i) Se 100%; (ii) Se 90% Te10%; and (iii) Se 80% Te 20%. Correspondingly, the occupancy of Te/Se(1) sites is (i) Te100%; (ii) Te90% Se10%; and (iii) Te80% Se20%, so that atomic ratio of Te and Se is 1:1 in total. The calculated patterns are presented in Fig. 4b. In the 80% ordering pattern, the intensities of 003 and 006 are almost same, but the experimental data are not. The value of *I*_006_/*I*_003_ is used for determining the Se occupancy accurately and the Te/Se(2) sites are considered to be approximately 95% occupied by Se atoms (inset in Fig. 4b). This value is in agreement with our ABF-STEM image simulation result. Also, the degree of Se ordering is in the same range of that reported in BSTS system^52^. As the Fermi level of PbBi_2_(Te,Se)_4_ is reported to cross the bulk conduction band^38^ and above the bandgap, lowering the Fermi level by Sb doping is needed to achieve bulk insulation. In the last section, Se ordering in Pb(Bi,Sb)_2_(Te,Se)_4_, which has a large bulk bandgap and high potential for true bulk insulation, is investigated from the pXRD analysis.

In Fig. 4c, the pXRD data of Pb(Bi_1-x_Sb_x_)_2_(Te_1-y_Se_y_)_4_ (*x* = 0.8, *y* = 0.5) and the simulated pXRD data are presented. In the case of Pb-BSTS alloy, there is single phase region of target compound in Bridgman batch, so it is possible to single out only Pb-BSTS phase. Among the three possible Se geometries, the experimental data most fit the inner Se ordering and are completely distinct from others. Lastly, we would like to discuss the physical origin of the inner Se ordering. In general, atoms with high electronegativity (EN) tend to make chemical bonds with atoms with low EN, because it is energetically favorable^53^. In PbBi_2_(Te,Se)_4_, Se has the highest EN (=2.4). The EN of Te (2.1) is lower than that of Se, and those of Pb (1.8) and Bi (1.9) are even lower. In the layer structure shown in Fig. 1, the site of Te/Se (2) has three bonds with Pb and three bonds with Bi while the site of Te/Se (1) has three bonds with Bi and three bonds with Te/Se. Then, when Se and Te separately occupy the sites (2) and (1), respectively, i.e., in the inner Se ordering, the total energy should be the lowest. As the EN of Sb (1.8) is almost the same as that of Bi, Sb doping should not break the inner Se ordering. This material has the largest bandgap in the inner Se geometry^38^, and the Se ordering structure is considered to suppress the formation of antisite defects^21^. Therefore, if we can finely tune the chemical potential, truly bulk insulating samples, which is ideal for surface states utilization for spintronics should be obtained.

## Conclusion

In this study, by combining state-of-the-art Scanning Transmission Electron Microscopy (STEM) and powder-XRD (pXRD) analysis, we studied the Se atoms distribution in Pb(Bi,Sb)_2_(Te,Se)_4_ TIs. In the STEM study, we directly observed the inner Se ordering of PbBi_2_(Te,Se)_4_ TI. The pXRD study revealed the strong tendency of Se atoms to occupy the inner site, and that quantitatively nearly 95% of Se atoms occupy the inner Te/Se(2) site. A qualitative view of local non-stoichiometry such as point defects is also presented from STEM observations. Additionally, we found that this ordering also occurs in Pb(Bi,Sb)_2_(Te,Se)_4_, which enables the bulk insulation by Fermi level tuning changing Bi/Sb ratio. These findings will pave the pathway to realize more bulk insulation in Pb-BSTS TIs than BSTS TIs.

## Method

### Synthesis

As described in earlier report^32^, PbBi_2_(Te_1-y_Se_y_)_4_ and Pb(Bi_1-x_Sb_x_)_2_(Te_1-y_Se_y_)_4_ crystals were grown using the Bridgman method. First, a mixture of high- purity (6N) Pb, Bi, Sb, Te and Se elements, in the stoichiometric molar ratio, was sealed in an evacuated quartz ampoule. Then, it was melted and homogenized at 800 °C, followed by water-quenching. Subsequently, the ingots were subjected to the Bridgman method for crystal growth. The ampoule translation speed was set at 2–2.5 mm/h. The temperature gradient in the furnace was approximately 25 °C/cm around the position of the liquidus temperature.

### Specimen preparation and STEM observation

To investigate the Se distribution, TEM samples of the PbBi_2_(Te_1-y_Se_y_)_4_ (*y* = 0.5) for [100] cross-sectional observation were prepared using conventional methods including mechanical polishing, dimple grinding and Ar ion polishing. STEM observation was performed using a 300-kV aberration-corrected electron microscope (Thermo Fisher Scientific, Titan^3^).

## Supplementary information

Supplementary Information.
