# Supplementary material for: Evidences of inner Se ordering in topological insulator PbBi2Te4-PbBi2Se4-PbSb2Se4 solid solutions
Source: Sci Rep. 2020 May 14;10:7957. doi: 10.1038/s41598-020-64742-6 (PMC7224274; doi:10.1038/s41598-020-64742-6)
Supplement: Supplementary file 1 — Supplementary Information. [file 41598_2020_64742_MOESM1_ESM.pdf]

Supplementary Information:

Evidences of inner Se ordering in topological insulator $PbBi_2Te_4$-$PbBi_2Se_4$-$PbSb_2Se_4$ solid solutions

Yuya Hattori[1], Yuki Tokumoto[1], Koji Kimoto[2], Keiichi Edagawa[1]

[1]*Institute of Industrial Science, The University of Tokyo, Komaba, Meguro-ku, Tokyo 153-8505, Japan*

[2]*National Institute for Materials Science (NIMS), Tsukuba, Ibaraki 305-0044, Japan*

Contents

S1. Microstructure and phases of $PbBi_2(Te_{1-y}Se_y)_4$ and $Pb(Bi_{1-x}Sb_x)_2(Te_{1-y}Se_y)_4$

Figure S1. EPMA Pb mapping of $PbBi_2(Te,Se)_4$ and $Pb(Bi,Sb)_2(Te,Se)_4$

Table S1. The composition analyses in $PbBi_2(Te,Se)_4$ and $Pb(Bi,Sb)_2(Te,Se)_4$

S2. The validity of Richardson-Lucy deconvolution for STEM images

Figure S2a. comparison of HAADF raw data and RL deconvoluted image.

Figure S2b. Large image of HAADF image

Figure S2c. Comparison of ABF raw image and RL deconvoluted one

S3. Effect of preferred orientation and antisite defects on pXRD intensity

Figure S3. Te/Bi intermixing effect to I_{006}/I_{003} ratio

S1. Microstructure and phases of $PbBi_2(Te_{1-y}Se_y)_4$ and $Pb(Bi_{1-x}Sb_x)_2(Te_{1-y}Se_y)_4$

For quantitative composition analyses, a melt-spun $Pb_{14.29}Bi_{5.71}Sb_{22.86}Te_{27.14}Se_{30.0}$ sample produced by a single-roller method was used as a composition standard for the ZAF conversion. Pb Mα1, Bi Mα1, Sb Lα1, Te Lα1 and Se Kα1 were used as characteristic X-ray for each element.

Fig. S1 shows EPMA-Pb mapping. Each phase is most easily distinguished when we choose Pb other than Bi, Sb,Te, Se for EPMA 2D-mapping. As is evident from Fig. S1, there are many phases: $PbBi_2(Te,Se)_4$ (phase A, blue arrow), $Pb_5Bi_6(Te,Se)_{14}$ (phase B, green), Pb(Te,Se) (phase C, red) and $(Bi,Sb)_2(Te,Se)_3$ (phase D, purple). Their detailed composition is listed in table S1. We can see that phase microstructure completely changes from (a) $PbBi_2(Te,Se)_4$ alloy to (b) $Pb(Bi,Sb)_2(Te,Se)_4$ alloy.

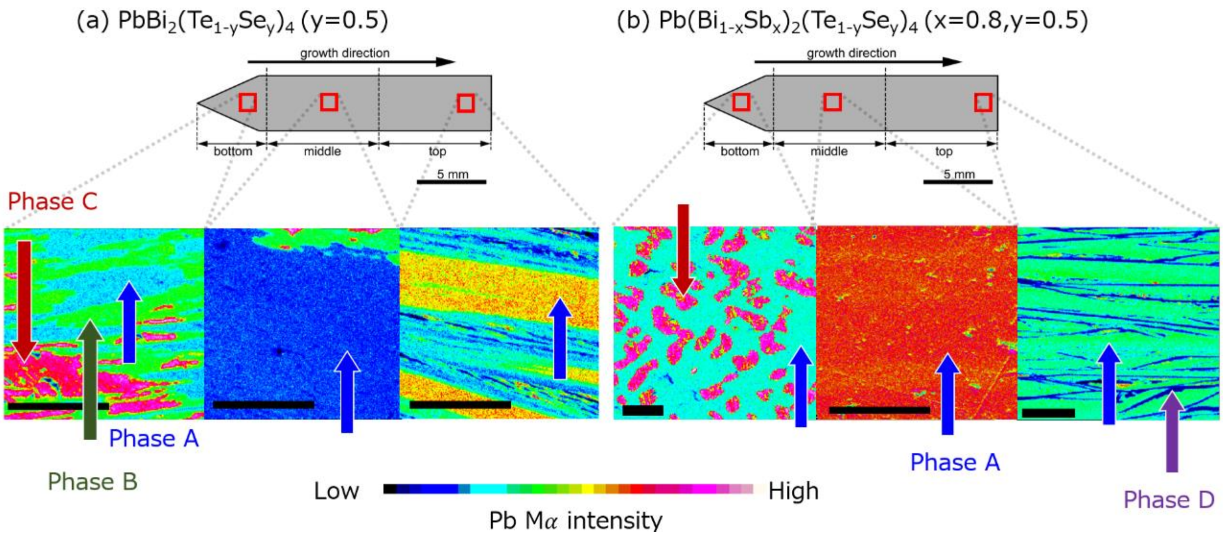

Figure S1. EPMA Pb mapping of $PbBi_2(Te,Se)_4$ (y=0.5) and $Pb(Bi,Sb)_2(Te,Se)_4$ (x=0.8, y=0.5). The size of black stick in the pictures is 200μm.

	Pb	Bi	Te	Se
Pb(Te,Se)	35.30	1.93	21.64	40.96
$PbBi_2(Te,Se)_4$	10.23	19.36	36.87	33.23
$Pb_5Bi_6(Te,Se)_{14}$	19.24	16.04	35.69	28.93

	Pb	Bi	Sb	Te	Se
Pb(Te,Se)	40.40	0.00	7.60	18.83	33.17
$Pb(Bi,Sb)_2(Te,Se)_4$	12.20	8.00	20.47	29.89	28.45
$(Bi,Sb)_2(Te,Se)_3$	1.20	3.29	33.51	33.59	28.39

Table S1. The composition analyses in $PbBi_2(Te,Se)_4$ (left) and $Pb(Bi,Sb)_2(Te,Se)_4$ (right)

S2. The validity of Richardson-Lucy deconvolution for STEM images

Here we applied Richardson Lucy deconvolution[1,2,3] for HAADF image to enhance the resolution (Fig. S2a and S2b). Ishizuka et al. utilized these algorithms to enhance spatial resolution in HAADF[4]. Under appropriate iterative process condition[4], we can effectively reduce the noise of raw data.

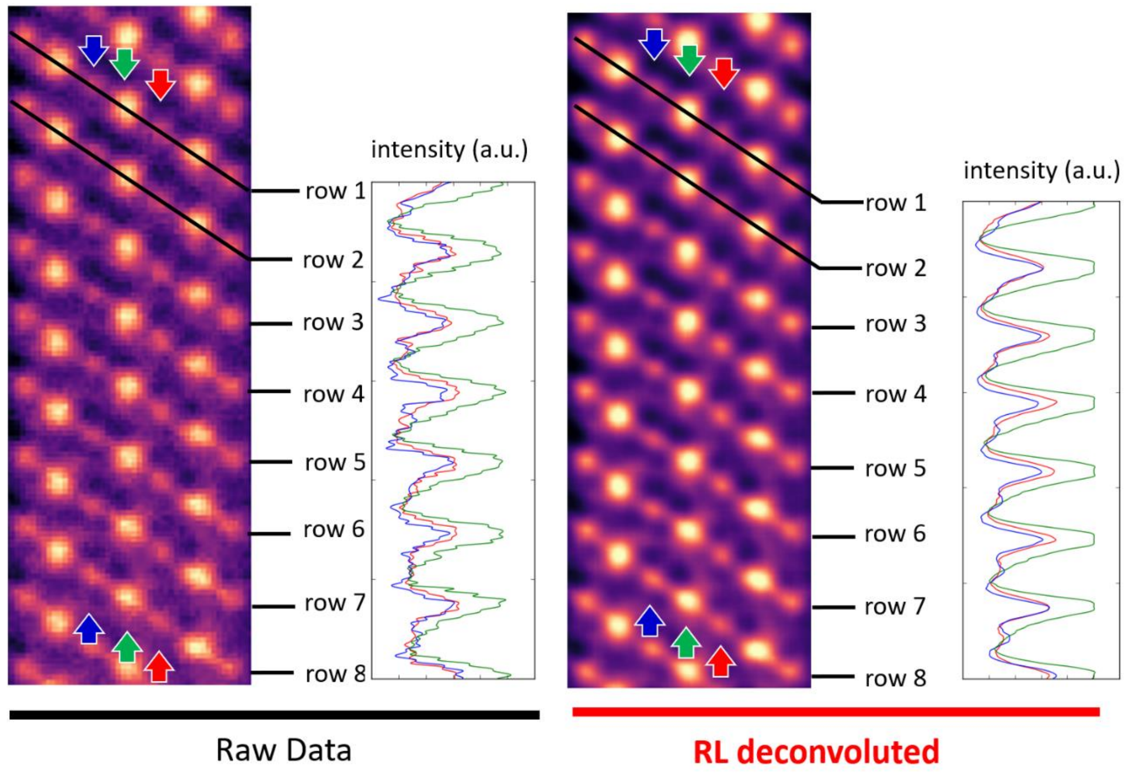

Figure S2a. Comparison of the HAADF raw image and the RL deconvoluted image.

Fig. S2a shows the intensity profile for each atomic sites, in which arrow color (blue, green, and red) and intensity line color are one to one corresponded. We can see the intensity fluctuation of inner Te/Se(2) columns in raw image (row blue and red). In the raw line profile, however, the intensity line profile is noisy and high intensity Se sites (red, row4 for example) and low ones (blue, row4) are hardly distinguished. In the deconvoluted line profile, however, row 4, 5, and 6 have both high and low sites in the same Se(2) inner sites. This tendency is also visible in raw image, so we can conclude that denoising was conducted in an accurate manner. We also find such fluctuation in other areas (Fig. S2b, yellow arrows), and seemingly they form

weak/high intensity pairs with next neighbor Te/Se(2) site across center Pb column.

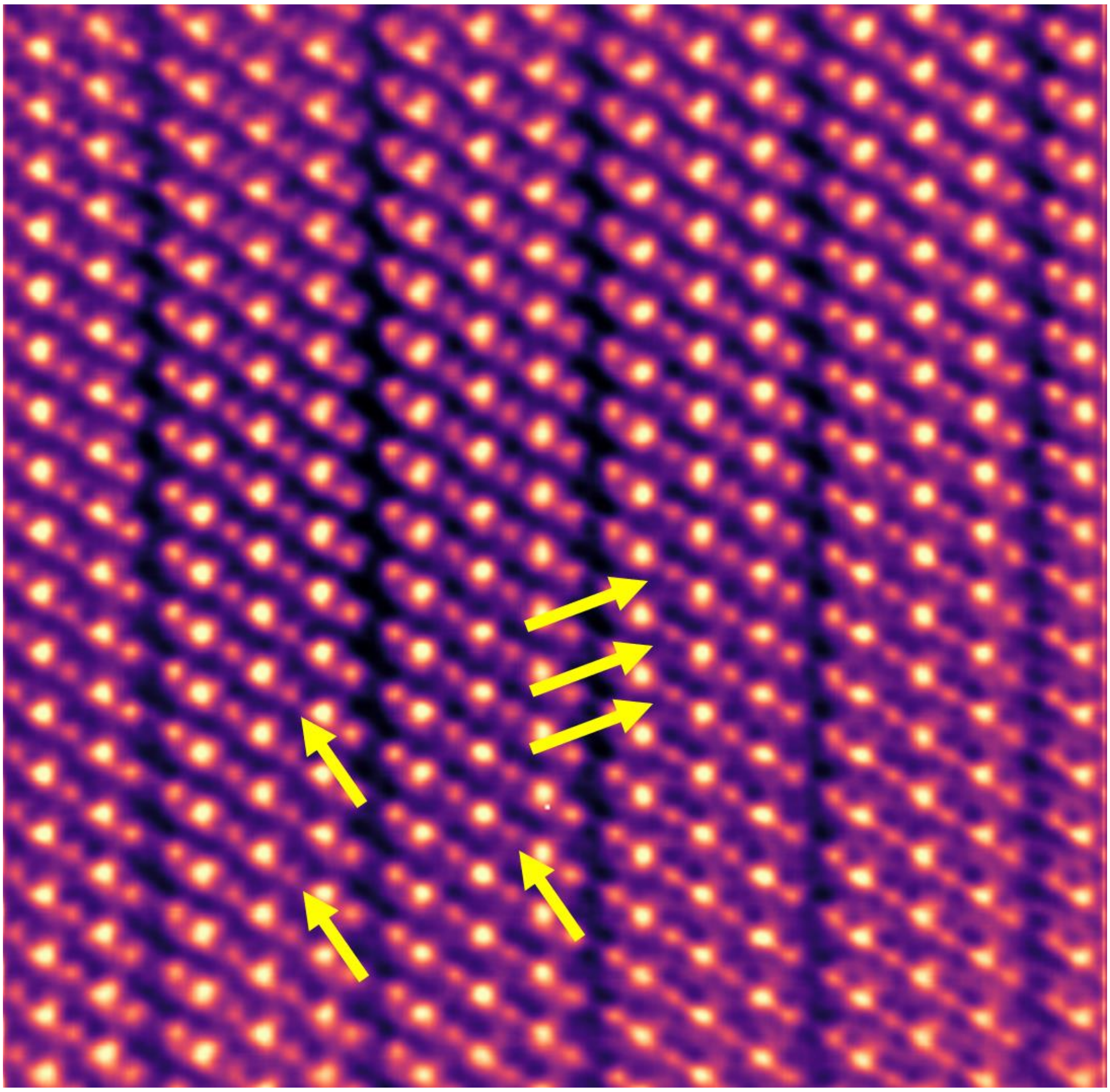

Figure S2b. A large image of HAADF image (RL deconvoluted)

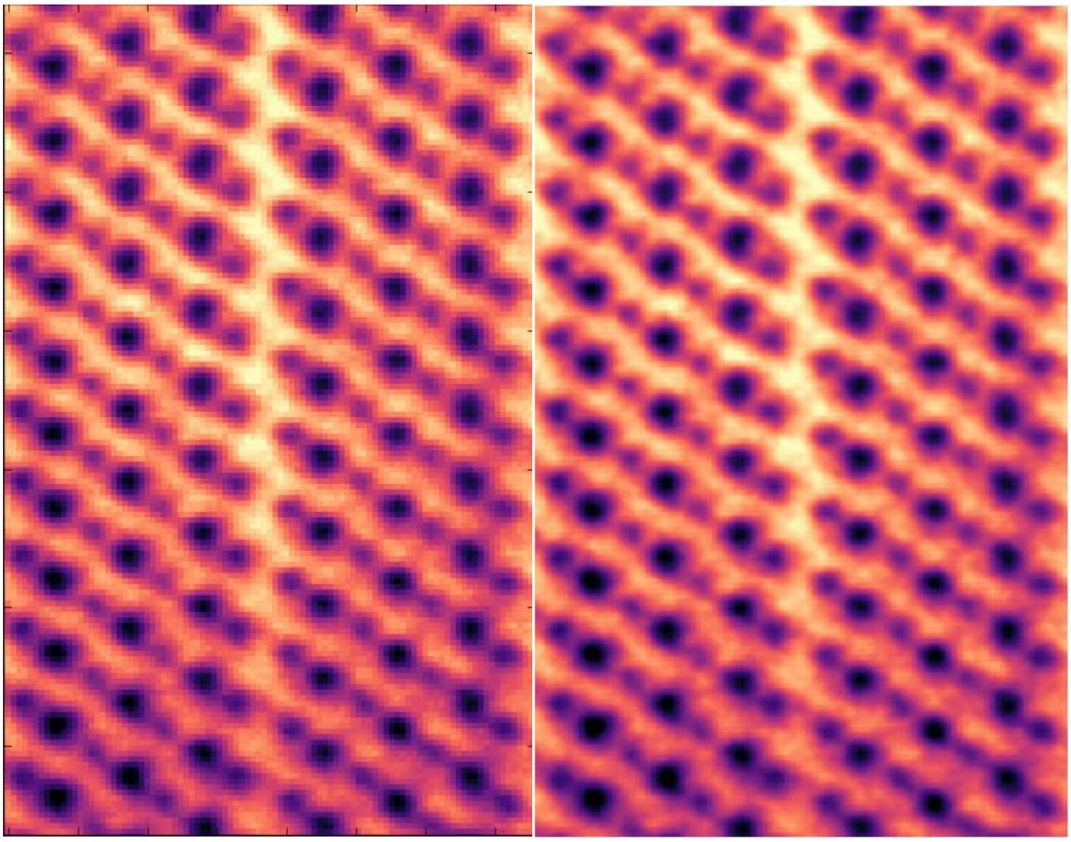

Figure S2c. Comparison of ABF raw image (left) and RL deconvoluted one (right).

Also, as for ABF image (Fig. S2c), we can see that the denoising process does not change its nature.

S3. Effect of preferred orientation and antisite defects on pXRD intensity

First, we compare the intensity from the same orientation plane; 003, 006, and 009. These belong to the same (00h) plane (cleavage plane of this compound); therefore, even when the preferred orientation occurs, the intensity ratio of these reflection does not change.

Second, according to the report of Shelimova[5], the dominant anti-site defects in $PbBi_2Te_4$ are V_{Te}, Te_{Bi}, Bi_{Pb}, which yield an n-type carrier. Bi_{Pb} does not affect the intensity in XRD measurements, and V_{Te} is thought to much fewer and hardly affect. The most effective antisite defect in the pXRD intensity is assumed to be the Te_{Bi} antisite defects. From the STM study of the Bi_2Te_2Se compound, the intermixing of Te and Bi rarely happens and ratio is less than 1%[6]. It should also be the case for Pb-BTS TI, then we calculated the pXRD of the worst case that affects the 00h intensity most: 10% Te migrate to the Bi sites, and 5% migration, comparing with the no migration case (Fig. S3). The more Te migration occurs, the higher the I_{006}/I_{003} value becomes. Considering the inset of Fig. 4b in the main text, this tendency may overestimate the Se ordering (for a perfect crystal). However, because the I_{006}/I_{003} value changes at most 3.78/3.08=1.24 times by the intermixing of Te and Bi. Experimental value of the $I_{006}/I_{003} = 2.17$ (Fig. 4b in the main text) should be reduced to $2.17/1.24 = 1.75$ at its worst case. However, Se occupancy in Te/Se(2) should be still around 90% (see Fig. 4b).

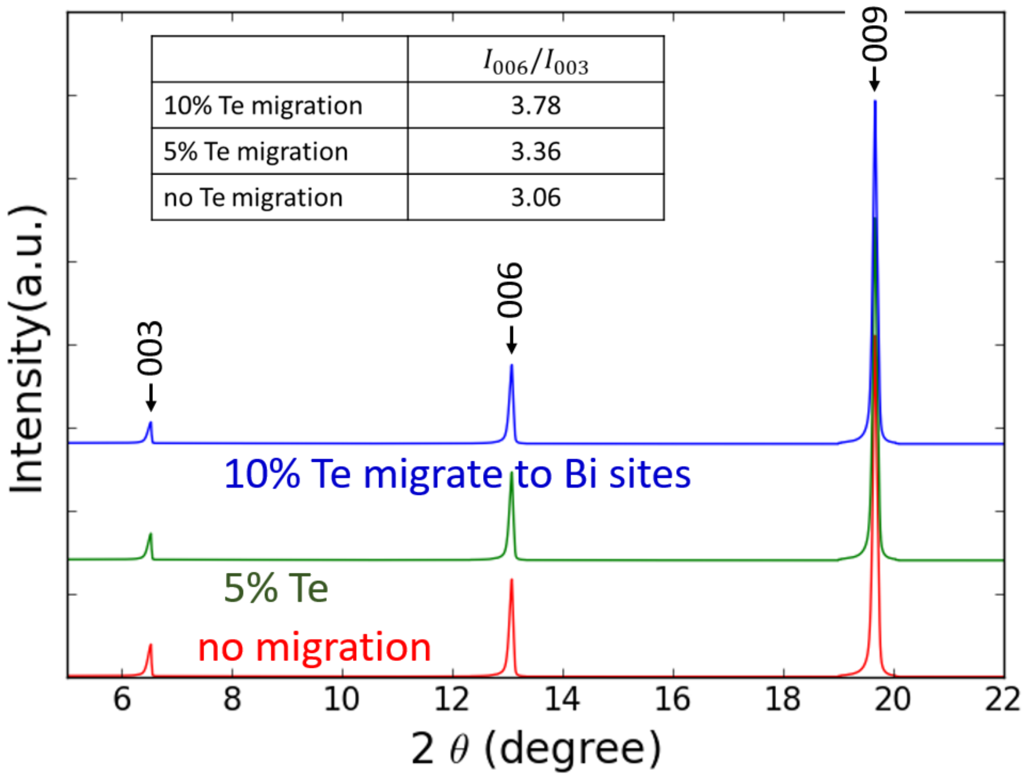

	I_{006}/I_{003}
10% Te migration	3.78
5% Te migration	3.36
no Te migration	3.06

Figure S3. The Te/Bi intermixing effect to the I_{006}/I_{003} ratio. The powder XRD simulation was conducted by RIETAN-FP[7]. As for an isotropic atomic displacement parameter (B), B is reported to range from 1.0 to 2.0 Å^2 in the compound of $PbSb_2Te_4$ and $PbBi_4Te_7$[5], and 0.4 Å^2 in Bi_2Te_2Se[8]. Considering Pb-BSTS is composed of all heavy atoms, we fix B= 1Å^2 for all atoms in this calculation.

References

1. Richardson, W. H. Bayesian-Based Iterative Method of Image Restoration*. *J. Opt. Soc. Am.* **62**, 55 (1972).
2. Lucy, L. B. An iterative technique for the rectification of observed distributions. *Astron. J.* **79**, 745 (1974).
3. van der Walt, S.; Schönberger, J. L.; Nunez-Iglesias, J.; Boulogne, F.; Warner, J. D.; Yager, N.; Gouillart, E.; Yu, T. Scikit-Image: Image Processing in Python. *PeerJ* **2**, e453 (2014).
4. Ishizuka, K. Deconvolution Processing in Analytical STEM: Monochromator for EELS and Cs-Corrector for STEM-HAADF. *Microsc. Microanal.* **11**, 1430 (2005).
5. Shelimova, L. E. *et al.* Crystal structures and thermoelectric properties of layered compounds in the ATe-Bi_2Te_3 (A = Ge, Sn, Pb) systems. *Inorg. Mater.* **40**, 451 (2004).
6. Jia, S. *et al.* Defects and high bulk resistivities in the Bi-rich tetradymite topological insulator $Bi_{2+x}Te_{2-x}Se$. *Phys. Rev. B* **86**, 165119 (2012).
7. Izumi, F. & Momma, K. Three-Dimensional Visualization in Powder Diffraction. *Solid State Phenom.* **130**, 15 (2007).
8. Jia, S. *et al.* Low-carrier-concentration crystals of the topological insulatorBi_2Te_2Se Shuang. *Phys. Rev. B* **84**, 235206 (2011).
